# Partnering With Teens With Past Suicide‐Related Crises: Development, Application and Refinement of Safety Procedures for Co‐Design

**DOI:** 10.1111/hex.70520

**Published:** 2026-02-09

**Authors:** Marisa E. Marraccini, Telieha J. Middleton, Lauren E. Delgaty, Ceren E. Iz

**Affiliations:** ^1^ School of Education University of North Carolina at Chapel Hill Chapel Hill North Carolina USA

**Keywords:** adolescents, co‐design, digital interventions, suicidal thoughts and behaviours, virtual reality

## Abstract

**Background:**

Growing interest in participatory co‐design approaches for digital mental health interventions necessitates attention to the development of safety protocols for safely partnering with individuals with suicide‐related risk. Historically, adolescents with suicidal thoughts and behaviours (STB) have been excluded from such approaches, discounting the importance of youth voice.

**Methods:**

This case study provides an overview of the development and use of safety protocols for participatory co‐design with adolescents with histories of suicide‐related crises who identify as ethnic‐racial minoritised or lesbian, gay, bisexual, transgender, queer or questioning (LGBTQ+). We also explored teen co‐designer perspectives on the safety protocols employed, using applied thematic analysis to identify themes informing co‐design safety for adolescents with suicide‐related risk.

**Results:**

No safety issues emerged during co‐design sessions, with the majority of adolescent distress levels remaining the same before and after meetings. Adolescent co‐designers endorsed many components of the safety protocol, with recommendations for improvements mainly addressing the need to adapt the protocol based on the unique needs of each teen.

**Conclusion:**

Future work can draw from lessons learned in this case study to enact participatory co‐design approaches that acknowledge and leverage the strengths and resilience of young people with STB.

**Patient and Public Contribution:**

Adolescents with histories of suicide‐related crises who identify as ethnic‐racial minoritised or lesbian, gay, bisexual, transgender, queer or questioning (LGBTQ+) were involved as partners in this study, which followed principles of participatory co‐design to improve a virtual reality intervention to enhance suicide prevention for hospitalised youth.

## 1 Introduction

Participatory co‐design approaches contribute critical information for improving interventions [1, 2, 3], with the central idea being that people with lived experiences expected to benefit from the approach ‘are best positioned to articulate these solutions’ [4, p. 6]. Researcher‐lived experience partnerships enhance and improve the research process, and, in turn, lived experience collaborators enjoy and benefit from the experience [1, 5, 6]. While numerous studies document partnerships with individuals with lived experience to build and test digital mental health interventions (DMHIs), adoption of these partnerships into youth suicide prevention research is limited [7, 8, 9]. Moreover, existing research involving partnerships with young people with suicidal thoughts and behaviours (STB) has primarily occurred with older adolescents (i.e., ages 16 or 17 and older) and young adults in the United Kingdom and Australia, without specific attention to youth with marginalised identities. Given that a common barrier to this type of work includes safety concerns for youth and researchers involved in these partnerships, the purpose of this paper is to describe one study's approach to developing and implementing safety procedures for partnering with youth with histories of suicide‐related crises in the adaptation of a DMHI in the United States and share the perspectives of ethnic‐racial minoritised and lesbian, gay, bisexual, transgender, queer or questioning (LGBTQ+) youth co‐designers participating in the study.

### 1.1 DMHIs for Preventing Youth Suicide

STB and emergency department visits for suicide‐related crises among adolescents in the United States have increased over the past decade [10, 11]. Approximately 20% of adolescents report seriously considering a suicide attempt, and 9% report having attempted suicide (CDC, 2024), with the number of paediatric hospitalisations for suicidal behaviours increasing by 31% over the past 10 years [12]. Because youth often turn to social media, technology and online tools when seeking mental health‐related information or help [13, 14], there is a pressing need for relatable and engaging DMHIs.

DMHIs, including online and app‐based forms of therapy and interventions, have been found effective for treating depression and anxiety [15, 16]. Unfortunately, DMHIs primarily encompass interventions developed using a ‘top‐down’ approach, in which experts determine the best method and content based on the evidence, with minimal attention to user/patient perspectives [4]. Given the variable rates of treatment utility among youth following a suicide‐related crisis [17], researchers must prioritise partnerships with young people to facilitate improved engagement and uptake of DMHIs [18, 19].

### 1.2 Participatory Co‐Design With Youth With Suicide‐Related Risk

Participatory co‐design includes several different participatory methods to move research beyond more traditional methods (in which end users voice ideas) towards a joint exploration of determining and developing solutions (in which end users hold power in decision‐making) [20]. Young people can be involved in co‐design, co‐creation and co‐production through a series of approaches (e.g., co‐design workshops, focus group interviews, card sorting tasks, friendship interviews, usability testing, mobile diaries, etc.) [18]. Cautions against co‐designing interventions and strategies with individuals with STB have centred around fears and concerns for participant safety (i.e., beliefs that participation will cause distress and result in STB) [6, 8, 9]. Although this belief perpetuates stereotypes that individuals with suicide‐related risk are fragile, vulnerable and in need of protection [6], ethics committee members have voiced concerns about the potential for research to exacerbate STB and the competency of these patients to provide consent [21]. Others have expressed concerns about a lack of researcher training and minimal resources to engage in co‐design procedures with these participants safely [8].

Fortunately, several frameworks for safely engaging in co‐design partnerships with youth with lived suicide‐related experiences set the stage for these co‐design practices [22, 23]. Webb et al. (2023) published guidance on the topic based on an expert Delphi consensus method that involved researchers and young people (aged 15–30 years) with lived experiences of suicide and/or self‐harm, previously having participated in research, and not reporting STB over the past 2 weeks [23]. Guidelines list multiple steps for safely engaging in lived experience partnerships (preparation, safety and well‐being, and evaluating involvement) and offer tips for young people to determine their own readiness to participate.

A small number of studies have successfully employed co‐design approaches with youth addressing suicide‐related content without any adverse events [9, 19, 24], but only a few have focused on minor‐aged adolescents with a history of STB or self‐harm [7, 25]. Findings from studies describing implications for safety during co‐design (see Table 1) emphasise the importance of clear communication between researchers and co‐designers that clarifies each member's role using appropriate language, well‐thought‐out safety procedures to handle distress, and ongoing evaluation to improve practices. To date, minimal research has provided a detailed description of how safety protocols have been developed, administered and received among adolescents with recent suicide‐related crises, and none have focused specifically on the perspectives of youth with marginalised identities.

**Table 1:** Studies of participatory co‐design approaches conducted with young people.

Reference	Intervention	Study overview (location)	Safety procedures	Key safety takeaways
[6]	#MyGPguide, which provides support in visiting a general practitioner for young people with lived experience of self‐harm and suicidality	Youth advisory group involving 18–25 year‐olds with lived experience or strong interest in mental health (the United Kingdom)	Established ground rules for involvement (i.e., confidentiality and its limits, acknowledging and respecting expertise across roles; listening respectfully; allowing time and space for sharing; turn‐taking) Consulted young people about perspectives on safe involvement Wellness or safety plan One‐on‐one debriefing session Set the parameters of sharing Assured that no members felt coerced or pressured to participate Flexibility to allow all members to feel safe and engage as appropriate	Youth Participation Lead (YPL) to be a consistent member working with youth Importance of establishing ground rules for involvement Need for proactive safety procedures for engagement Need for support and training for researchers Importance of clear, reciprocal and supportive communication procedures
[25]	Web‐based conferencing technology for focus groups with young people with STB	Online focus groups with young people ages 16–25 years (mean age 21.3) with lived experience of suicidal ideation, excluded those with schizophrenia or related psychotic disorder, suicidal thoughts in the past 2 weeks, or suicide attempt in the past month (Australia)	General Risk Management Procedures:	Five considerations for work such as this in the future:
			Reminding that a clinician is available Messaging the moderator about distress Setting ground rules (confidential reminders, limiting personal information shared)	Considering the health status and technology literacy of participants during recruitment; Reminders that participants do not need to discuss anything difficult or painful; Establishment of safety procedures involving clinicians and allowing withdrawal, with follow‐up check‐ins as needed; Reminders to value diverse ideas and opinions and reinforcing equal opportunity for participation and Ending sessions with self‐care‐oriented strategies
			Identification and Management of Risk:	
			Self‐report/disclosure Breaks from meeting with an additional facilitator in touch Check in at the end of the session Follow‐up call the following day Contact of carers/health professionals as needed	
[9]	#chatsafe campaign strategy for suicide prevention in Australia	Participatory co‐design process with 134 young people (ages 16–25; no eligibility criteria based on suicidal thoughts and behaviours) (Australia, Orygen)	Collaboratively developed wellness plan addressing emergency contact details Risk and management protocol employed Psychologist available during and after each workshop Brief evaluation of safety following meetings, asking young people if they found the workshop distressed them, and if they wanted to discuss their experience with a research team member Structure of the sessions included a warm‐up, co‐design activities, and evaluation and cool‐down	Safety concerns (young people expressing distress or suicidal thoughts and behaviours as a result of workshops) emerged, necessitating evaluations of participation in future
[24]	Self‐monitoring app to manage symptoms of depression	Human‐centred co‐design studio methodology conducted with 8 young people (aged 18–25) with a history of depression and/or STB (individually and then collaboratively), also involving clinician perspectives. Inclusion criteria restricted people with suicidal thoughts and behaviours in the previous 3 months (Australia, Orygen)	Youth collaborated on a wellness plan before completing sessions. ‘Robust procedures for ensuring the safety and well‐being of participants were developed and implemented’	Not reported
[7]	CaTs‐App, which is a digital version of a previously developed Card‐Sort Task used for research about self‐harm	Several different co‐design workshops with young people aged 17–24 with a history of self‐harm (the United Kingdom)	Priorities:	Sustained structure with dedicated patient and public involvement members that is balanced with outside participants (given young people age out)—time commitments not always consistent (as expected) Resource‐ and time‐intensive processes for both researchers and participants More work dedicated to documenting efforts for patient and public involvement is needed Need to be able to consider and address the risk and put in appropriate safeguards for this type of work Excluding those in recent crises for safety comes with its own negatives (those excluded may benefit most and best reflect those that the intervention addresses)
			Meaningful, safe, responsible and engaging collaboration Early and sustained involvement Inclusion of diverse and marginalised voices Outcomes and evaluation Guided by design models/principles	
			Four objectives of patient and public involvement:	
			information and clarity accessible and collaborative ways of working prioritising safety Ensuring young people are recognised for their contribution.	
[26]	LifeBuoy (a smartphone app to help young people manage suicidal ideation)	Two co‐design workshops involving collaborations with 3 young people (aged 19–24) having previously experienced suicidal thoughts, identified from the 16 interviewee participants (18–25) completing qualitative interviews during the discovery phase, selected based on the depth of their previous interviews (Australia)	Opportunities for questions in advance of the workshops Anonymity during workshops (advisors turned their cameras off and used pseudonyms) during remote Zoom meetings. Encouragement to inform researchers (verbally or by private chat) if feeling distressed or wanting to break/stop/withdraw Immediate access to a clinical psychologist and contact by a psychologist within 48 h if distress emerges Reminders of Lifelines Australia (24‐h crisis contact) and primary care professional	No safety concerns emerged

### 1.3 The Present Study

This paper presents one study's approach to developing and implementing safety procedures for partnering with youth with lived experience, including LGBTQ+ and ethnic‐racial minoritised co‐designer perspectives on these procedures. Our case study is part of a larger project developing and piloting co‐designed vignettes for a VR‐based intervention, Practice Experiences for School Reintegration (PrESR), which augments inpatient treatment for adolescents hospitalised for suicide‐related crises [27]. Specific aims of this study are to: (1) develop, apply and refine safety protocols for partnering with youth with STB based on community and youth feedback and (2) prioritise marginalised youth voices in co‐design activities and explorations of safety protocols.

## 2 Materials and Methods

### 2.1 Safety Protocol

The initial safety protocol was developed based on study goals and existing guidance [6, 23]. Our team first reviewed relevant literature to develop safety protocols, establish a recruitment approach and determine eligibility criteria. We then sought feedback from local clinicians working with racial‐ethnic minoritised and LGBTQ+ teens with a history of suicide‐related crises, refining the protocol based on their feedback. Specifically, co‐design protocols, safety protocols and study procedures were revised iteratively—we updated the protocols in track changes based on clinician comments and sense checked ideas with subsequent clinicians providing feedback. Examples of changes include eligibility criteria that specified timing of the most recent crisis and therapy/counselling requirements depending on that timing, confidential methods for sharing ideas during group meetings, check‐in/check‐out procedures for monitoring safety, and suggestions for approaches to ensuring meetings were inclusive to youth with different engagement styles (e.g., having silent/optional ice breaker activities at the start that did not require sharing out). An abbreviated version of the safety protocol is shown in Table 2 (see Supporting Information for the complete version).

**Table 2:** Abbreviated overview of safety protocol for participatory co‐design with youth with suicide‐related crises.

Stage: Consideration	Description
Preparation:	
Community partner feedback	Community feedback from relevant members (i.e., experience working with youth with suicide‐related risk, as well as youth potentially marginalised due to their identities) to inform final safety protocols
Eligibility criteria to enhance safety	Eligibility criteria that prioritise *both* representativeness and readiness of co‐designers
Recruitment process	Opportunities for multiple contacts using different methods and approaches for considering the readiness of research Clearly specified lived experiences requirements (emergency encounter for suicidal thoughts and behaviours between the past 6 months and 2 years). Reminders that research is not a replacement for professional support and reinforcement of the importance of ongoing treatment and psychological care.
Meeting format(s)	Individual meeting with the researcher(s) before attending any group meetings to build rapport, address stressors and collaborate on a safety plan. Group sessions include at least two trained researchers/clinicians and include inclusive and diverse activities, opportunities to discuss concerns privately, and check‐in/check‐out procedures for monitoring distress. ∘ Initial meeting includes coming to a consensus on group rules and setting expectations for roles If possible, consider separate groups based on age or identity
Check‐ins between meetings	Reach out to youth to check in about safety and well‐being within a week of meetings using the preferred method
**Evaluations**	Provide co‐designers an opportunity to give formal feedback and also evaluate efforts through observations and processes throughout participation
**Safety and well‐being protocols**	Collaboration on a safety plan before group meetings Minimise risk, and plan for how to handle concerns that will emerge Monitor distress and offer calming strategies as needed Share ‘Review Tips’ from *Guidelines for Involving Young People with Lived and Living Experiences of Suicide in Suicide Research* (pp. 18–19).

### 2.2 Procedures of Study

This study was reviewed and approved by the Institutional Review Board (IRB; #23–0388). Teen co‐designers, meeting regularly to brainstorm and develop ideas for additional VR‐based vignettes, included seven adolescents who sought emergency services for a suicide‐related crisis.^1^ Sessions were held virtually in two different multi‐week periods. Following the first round of meetings, a semi‐structured ‘check‐in’ interview was completed individually with six co‐designers partcipating in the first multi‐week period to discuss feedback about safety protocols and procedures.

#### 2.2.1 Participants

Final eligibility criteria for adolescent co‐designers participating in virtual group meetings were: (1) past 2‐year emergency department encounter for STB, with most recent encounter occurring at least 6 months ago; (2) aged 13–19; (3) ability to speak, read and understand English sufficiently to complete study procedures; (4) consent of a parent/legal guardian (provided in English or Spanish); (5) adolescent assent; (6) self‐identification as (a) racial‐ethnic minoritised and/or (b) LGBTQ+; and (7) engagement in therapy or counselling depending on the timing of most recent crisis. Exclusion criteria included self‐report of current psychotic symptoms or intellectual disability.

#### 2.2.2 Recruitment Procedures

Adolescent co‐designers and sense checkers were recruited in one of two ways: (1) phone calls to parents/legal guardians (hereon referred to as parents) or adolescents 18 years or older who were identified based on medical records of patients previously seeking help for a suicide‐related crisis or (2) flyers posted at local organisations serving adolescents in the community (e.g., LGBTQIA+ centres). Recruitment of adolescents via identification from medical records involved a sequential approach in which we: (1) identified potentially eligible adolescents; (2) reviewed their medical chart to confirm eligibility (if possible, not all eligibility criteria were viewable in medical charts); (3) mailed an opt‐out letter to the family explaining that a researcher would call them about eligibility for a research study, providing them the opportunity to contact the researchers to opt out of being recruited; and (4) followed up by phone to seek parent permission (before speaking with and reviewing assent procedures with minor adolescents) or adolescent consent (in cases when adolescents were 18 years or older). All participants in this phase of the study were under the age of 18, with permission provided by parents and informed assent completed by adolescents.

#### 2.2.3 Co‐Design Group Workshops

Two rounds of virtual co‐design sessions were held, including six adolescents in December 2024 and five adolescents in March 2025. Each round included four biweekly or weekly virtual meetings. Each group session included a minimum of two trained facilitators, both with clinical training related to working with youth with suicide‐related risk. The first author, a licensed psychologist with school psychology training, facilitated each session, with support from the second author, a doctoral school psychology student with a Master of Arts (MA) in counselling. Examples of activities, which were conducted using Miro as a collaborative whiteboard, included brainstorming stressful events (verbally and in writing), card‐sorting ideas (using bright colours and simple words), group prioritisation via dot voting or chats/verbal inputs to co‐facilitators, dialogue building (involving reading and acting out scenarios), and storyboard building. Because workshops included adolescents between ages 13 and 17, they included multiple modalities for engaging and providing feedback appropriate for developmental and cognitive differences to prioritise safe group dynamics. For example, co‐facilitators pulled some participants into one‐on‐one break‐out rooms to troubleshoot or provide additional or more detailed directions, and while some participants engaged directly in Miro, others shared their ideas verbally or by chat messages with co‐facilitators to input into Miro for them.

#### 2.2.4 Measures

##### 2.2.4.1 Demographics

Demographic questions addressed several characteristics, including race, ethnicity, gender and sex (see Supporting Information S1 for specific measures).

##### 2.2.4.2 Safety

###### 2.2.4.2.1 Protocol Deviations

The researchers documented and reported protocol deviations during the co‐design process according to standard IRB procedures. The number and type of protocol deviations are reported to inform safety protocol fidelity and compare risk versus benefits to this study.

###### 2.2.4.2.2 SUDS

**Figure 1:**
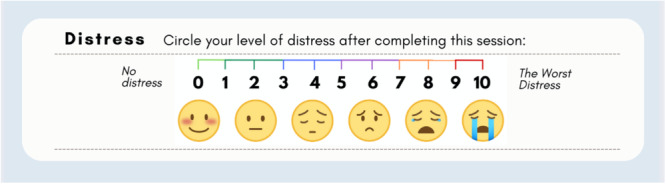
Subjective Units of Distress Scale. Artwork developed by Laena J. Marraccini.

Co‐designers were taught to use Subjective Units of Distress (SUDS) to self‐assess their level of distress on a scale from 0 to 10 [28] at the start and end of every co‐design meeting (see Figure 1). Before participating as a group, co‐designers shared their typical distress levels to inform an expected baseline for meetings. Co‐designers also identified the level of distress signalling their ‘danger zone’ or need for immediate support.

###### 2.2.4.2.3 Check‐In Interviews

The first author conducted individual, semi‐structured interviews with six co‐designers participating in the first round of co‐design sessions. The interview guide addressed five topic areas of perspectives, feedback and input: (1) being involved in the project; (2) new ideas since the last meeting; (3) the meeting set‐up and experience; (4) teen involvement in research; and (5) the safety protocols employed. The final area of the interview included a collaborative activity using Miro in which adolescents viewed a virtual whiteboard showing safety protocols and provided ideas and feedback. Note that for this study, we do not share findings from topic area 2, as it was focused on intervention development.

Interviews were audio recorded and lasted between 18 and 48 min. A trained research assistant documented notes during the sessions. The interviewer completed a debrief summary at the conclusion of each interview to identify the overall tone of the conversation, any disruptions or concerns, and key ideas expressed across categories.

#### 2.2.5 Data Analysis

We calculated average change scores in SUDS from the start and end of every co‐design session to explore participant safety. Audio‐recorded interviews were transcribed, reviewed for accuracy and redacted of identifying information. The final transcripts were entered into NVivo qualitative data analysis software (QSR International, 2022), and we conducted applied thematic analysis (ATA) to identify themes and categories relevant to our aim of prioritising marginalised youth voices in co‐design activities and explorations of safety protocols. ATA integrates multiple qualitative approaches based on several theories (e.g., inductive thematic analysis and grounded theory) to systematically explore qualitative data [29].

The second and third authors developed, applied and iteratively refined the coding structure. The researchers maintained an audit trail of changes to the coding structure to ensure transparency. To ensure credibility, each researcher first independently coded the transcript, meeting to come to a consensus across codes. The first, second and third authors then reviewed the final codes related to the research aims and identified themes and overall ideas related to co‐design activities and safety protocols, selecting illustrative quotes to enhance transferability.

#### 2.2.6 Reflexivity

Reflexivity, defined as the ‘critical awareness, acknowledgement, and questioning of the ways in which researchers' own attitudes or beliefs shape data collection, analysis, and interpretation’ [29, p. 282], is salient to both qualitative research methods and participatory co‐design approaches. We considered the ways in which power influenced not only our data analysis and findings, but also the development of our rapport with co‐designers and our ability to meaningfully partner with young people. In both group and individual sessions, we aimed to have transparent conversations with youth that acknowledged their role in this study as a partner, but also the boundaries in place to maintain a safe, welcoming and authentic environment for this study. The research team held weekly meetings to discuss these issues, with special attention towards the tension between empowerment and safety. It was through these reflexivity activities and processes that the aims of this case study, addressing teen perspectives of safety protocols and conducting individual interviews inclusive of this topic, were crafted.

## 3 Results

### 3.1 Sample Characteristics

Adolescents participating in virtual co‐design sessions included seven teens (aged 13–17) identifying their race, ethnicity, gender or sexual orientation in a way that can be considered ethnic‐racial minoritised and/or LGBTQ+. Data from multiple questions addressing race were reviewed for consistency, with only one person identifying their race differently across questions. Co‐designers identified their race as white (*n* = 5), Black or African American (*n* = 2); their ethnicity as Hispanic or Latine (*n* = 1) or non‐Hispanic or Latine (*n* = 6); their sex and gender as boy/man (*n* = 1), girl/woman (*n* = 4), in some other way (*n* = 1) and unsure if transgender (*n* = 1); and their sexual orientation as bisexual (*n* = 1), pansexual (*n* = 2), gay or lesbian (*n* = 2), heterosexual or straight (*n* = 1), and other (‘I like what I like’, *n* = 1).

### 3.2 Protocol Deviation

Our team made one protocol deviation during study procedures related to participant safety. Recruitment procedures delineated different eligibility criteria for co‐designers and sense‐checkers, who were identified using the same initial recruitment method, and then screened for eligibility separately. The protocol deviation included one adolescent participating as a co‐designer, despite an encounter for a suicide‐related crisis within the past 6 months. During recruitment, this adolescent was screened correctly, but then invited to participate as a co‐designer by mistake.

Upon discovery of this deviation, our team immediately contacted the family to explain our error and invite the teen to participate as a sense checker. We also reached out to the teen's therapist and notified the IRB of the protocol deviation. The family and teen shared no concerns related to their participation, and review of the teen's distress levels before and after each group meeting revealed no changes in distress (their SUDS score stayed at zero at the beginning and end of each meeting). No adverse events or serious adverse events occurred in relation to this deviation, and the teen continued to participate in the study as a sense checker. Because the teen had participated in two co‐design sessions, they were invited to complete the qualitative interview.

### 3.3 Distress

Average changes in distress levels based on SUDS across sessions for each participant ranged from −1.0 to 0, resulting in an overall average of −0.35. Seven participants completed a total of 37 sessions (ranging from three to eight per participant). Distress increased in only one instance (by one‐point, from zero to one, indicating increased engagement); distress levels stayed the same in 26 instances; and distress levels decreased by between 1 and 3 points in 10 instances. Taken together, changes in SUDS suggest comparable or improved levels of distress following teen participation in co‐design activities.

### 3.4 Qualitative Feedback

#### 3.4.1 Project Involvement

Co‐designers' reflections about their involvement revealed two themes: *Safe, But Sensitive* and *Positive, But Quiet*. The theme *Safe, But Sensitive* refers to participant descriptions of a safe and caring environment, supported by, but also hindered by, the need to avoid sensitive or potentially upsetting topics. One participant explained, ‘I feel like you guys do like a bunch of, like, stuff to kind of prevent, like, anything bad from really happening’. Another shared, ‘I like the process of it. And it's not as triggering as I thought it would be. Just talking about everything. It's, like, there are people there that like, understand that, like I guess, environment’*.*

Yet, to maintain safety, participants also described feeling like they could not always engage in deeper discussions because of rules preventing them from sharing more intimate details about their experiences. Accordingly, conversations could feel like ‘walking on eggshells a little bit’.

The theme *Positive, But Quiet* relates to the general endorsements provided by participants about their experience as co‐designers, but also frustrations related to their peers' level of engagement. Several described their peers as quiet: ‘not really talking people—but they were still kinda' cool’. A couple spoke to variability in levels of engagement, with one feeling pressure to keep the conversation going and another describing frustrations related to their peers getting ‘sidetracked’. Despite these concerns, the majority of participants described their experience as enjoyable or positive.

#### 3.4.2 Activities and Meeting Set‐Up

Most participants described the co‐design activities, as well as the structure and set‐up of the meetings, positively, and also offered ideas for improvement. General endorsement related to the overall structure of the meetings and the appropriateness of engaging in multiple methods of soliciting feedback. Participants described appreciating aspects of the set‐up, like the consistency of the meeting structure, the compressed time period of the meetings and the one‐on‐one meetings in advance of the group meetings:

I think that was a good idea because you could kind of tell like if they would be a good fit or not based on like if you're meeting them for the first time or not. And, also, just like, so you're not in a group setting and something happens and then someone gets triggered because they didn't know the protocol before.

Several described the activities as fun, especially the warm‐up and cool‐downs; several also felt that the activities could be confusing or reported that they had a hard time paying attention. Participants identified multiple ideas for improving the meeting structure, such as making it more personal, splitting participants into older and younger groups or groups with different foci (e.g., those interested in getting to work vs. those interested in socialising), and holding in‐person meetings.

#### 3.4.3 Teen Involvement in Research

All participants described teen involvement in research as important, with their reflections revealing two themes: *Teen Involvement to Improve Outcomes* and *Readiness of Teens and Researchers for Partnerships*. The theme, *Teen Involvement to Improve Outcomes*, describes the rationale for needing to include teens in research, such as providing teens the opportunity to help other people like themselves (‘I wanna' be a part of it, the research thing, and help other teens or other people come like come through, like how I came through’), leveraging teen expertise to ensure relevancy of interventions or approaches (‘I feel like they just have more expertise in that situation because it's like—it's first‐hand experience of like, social—social settings and like the feelings that come up in those settings’), and to minimise ‘lines between generations’ and ensure ‘involvement from everybody who's affected’.

The theme, *Readiness of Teens and Researchers for Partnerships*, addresses how both researchers and teens need to ensure they are ready to collaborate. One participant described how researchers should be prepared to speak in simpler terms and present materials in an engaging way, and another reflected on a previous research experience, describing how they (as a teen) may not have been ready to participate in research at that time in their recovery. Another, however, recommended to other teens: ‘maybe just to go for it, honestly’.

#### 3.4.4 Safety Protocols

Results pertaining to safety protocols are presented separately for protocols employed in advance of participation (eligibility criteria and recruitment) and during participation (e.g., check‐ins, distress monitoring, etc).

### 3.5 Protocols Employed in Advance

After providing an overview of the requirements for participating in the study, nearly all participants endorsed the eligibility requirements related to timing in recovery as reasonable. One shared, ‘After a certain point of not having any activity where you're at a certain point of instability, I guess then I think it's way safer and I guess easier for everybody to get information and give information and stuff’. Yet, a few also pointed to the need to consider differences within individuals when considering readiness for partnerships, given that some may be ready to participate earlier in their recovery than others:

But I feel like … there's different levels. Cause, like, you don't have to go too deep in for them to make it feel like it was a sensitive topic for them, but at the same time you don't wanna … you wanna, like get in their emotions and know how they felt at that time. But everybody's different in their own ways.

In particular, one participant shared how, for them, timing felt less important when determining their readiness to participate:

…You guys still have the rules set for like, ‘oh hey, don't, you know, bring up stuff that would trigger you’. I feel like everybody's pretty respectful of that. So even if they're still like recently getting out of, like, a hospitalization, I don't really think it matters to set like—like divide it, because nobody's really gonna' say anything.

Regarding our approach for screening participants based on race, ethnicity, sexual and gender identity, nearly all participants felt that using broad eligibility criteria to maintain the safety of kids not ‘out’ to their families was important: ‘I think that's really cool. Just because you didn't want to “out” people and I feel like that was, like, really considerate’. One teen described how providing some information, but not too much information, was critical: ‘And so I agree that you don't want to “out” them, but at the same time, I feel like how … like you *don't* want to say too much, but also you *do* want to say too much. So, it's kind of like in the middle type of thing’.

### 3.6 Protocols Employed During Participation

Participants were largely supportive and appreciative of the safety protocols employed, providing general endorsement of check‐ins between meetings, SUDS for monitoring distress (‘check‐ins and check‐outs at the beginning and end of both, I think are good because it helps kind of gauge reaction, I guess’) and implementation of group rules to maintain structure:

I definitely think you should, like, keep everything though, because I feel like, if you have like that and then, like, the confidentiality removed, like—I don't know—it would just kind‐of be like a shit show.

Although participants appreciated the researcher–co‐designer check‐ins, many were not available to connect with researchers in between meetings, therefore suggesting that researchers incorporate texting and encourage more frequent check‐ins when things are stressful:

I just think the texting thing, you should definitely add. And I feel like the frequency of check‐in is pretty good. I think—I don't—I mean, it wouldn't hurt to add like a little bit more, but like I'm not like be pushing that ‘cause I feel like it's pretty good, but if someone's like actually struggling, then, like, obviously they're gonna’ need more frequent like check‐ins.

A few also shared the importance of safety planning ahead of the meetings (albeit sometimes reluctantly, e.g., ‘It's like annoying, but it's not tedious. So, it's just like, “damn, I have to do this again. OK, whatever”’), with one commenting on their appreciation for anonymous identification, describing the combination of their favourite character and favourite colour as ‘fun’.

Regarding group rules, while a few felt like the rule about not sharing contact information was fine, a few others would have preferred the option to share their contact information with one another. One explained:

I feel like the contact info…. Like I understand it, but at the same time, like, you already have confidentiality and respect for privacy…hypothetically, if we could share contact information with each other, I feel like that would make us bond more and then therefore, like, wanna' talk more, because you get to know everybody, like, more than, like, just an hour over the screen. And I also feel like with that like people are like, ‘Oh, like I'm not supposed to share anything. Should I even be sharing like my name? Where I'm from?’

Several also described how the rules around minimising the sharing of stressful experiences dampened their overall experience and opportunity to connect with one another:

I think I've noticed that are some like restrictions to avoid triggering and stuff which I honestly feel like, if I, if people can, then it might be a good idea to embrace those so that we can, you know, research and solve. I guess the issues that are occurring, especially in kids who have been hospitalized for stuff like suicide and stuff—I think it's important to really talk about this stuff.

Finally, one participant shared ideas for handling this issue:

Just like use your brain, like just kind of like think about, ‘Oh, OK, I'm not going to share something super graphic or like an immense amount of details because that would be triggering to me, but I can share X and Y about this story….’ I feel like as long as you're not giving people, like, ideas on how to do stuff, it doesn't really matter. I mean besides, you know like triggering somebody. But even then, I feel like it might just be me, but I feel like a lot of people have, like, kind of like a—a like tolerance to like stuff like that.

## 4 Discussion

Given that safety is identified as a common barrier to engaging in participatory co‐design with teens with STB, this case study shared our process for developing and implementing safety protocols to engage in participatory co‐design with racial‐ethnic minoritised and LGBTQ+ adolescents with recent suicide‐related crises. Findings have implications for the ways in which safety protocols are developed and employed, strategies for engaging with youth during the co‐design process, and the importance of partnering with youth with lived experience for youth, research and improved practice.

### 4.1 Safety Protocols

Thoughtful safety protocols employed with fidelity, and also flexibility, appear critical to countering the risks and pitfalls of participatory co‐design with individuals with lived experience [6]. Review of co‐designer feedback and SUDS before and after each meeting supports that teen co‐designers with recent histories of suicide‐related crises could safely engage in co‐design sessions. Our teen co‐designers also largely agreed with the importance of the safety protocols employed in this study, yet still questioned implementation without consideration of variability in their own and others' individual experiences. Co‐designers were receptive to many of the standard safety procedures, such as protections of confidentiality related to sexual and gender identity, check‐in and check‐out approaches, and collaborative safety planning in advance of co‐design sessions; however, they were more critical of the safeguards preventing sharing potentially stressful information, cautions against sharing contact information between co‐designers, and criteria informing readiness for participation in the co‐design process.

Previous researchers have described setting parameters around the depth and type of information co‐designers can share [6, 25], yet co‐designers in this case study shared concerns about this approach dampening their comfort in disclosing anything. Given that our discussions of the safety protocol occurred after only four meetings, these concerns likely reflect the time required to build rapport across group members and employ feedback that encourages the appropriate content for discussions. Indeed, Babbage et al. describe the need for early and sustained engagement [7]. Our team was able to integrate this feedback into our subsequent meetings, attempting to redefine the researcher's and co‐designer's understanding around the type of information appropriate to share or not share during sessions. Consistent check‐ins with teen co‐designers were critical to inform this adaptation to our protocols during subsequent meetings.

The implications of co‐designer feedback about restrictions against sharing contact information are less clear. Although few studies have reported safety approaches related to sharing contact information among youth co‐designers with STB, at the most conservative level, Gan et al. described holding virtual meetings that required co‐designers to turn cameras off and use anonymous pseudonyms [26]. Hospitalisation practices do routinely restrict teen patients from sharing contact information, with previously hospitalised teens sometimes questioning these policies, despite reporting difficult social interactions [30] and ‘double‐edged friendships’ [31]. On the other hand, peer support appears to have protective effects against self‐harm among adolescents more generally, and when moderated appropriately, online support groups can have promotive effects for support and information sharing [32]. It remains unclear whether or not partnerships following hospitalisation should mirror these practices.

Teen perspectives on criteria pertaining to readiness to partner with researchers indicated the need to consider readiness on a case‐by‐case basis. Our protocol deviation offered a unique opportunity to understand one teen's perspective on participating despite a recent crisis, and their reflections (feeling ready to participate despite our study protocol technically precluding them) mirrored other participants' input (that readiness may be unique to each teen). Babbage et al. previously shared concerns related to exclusion practices given their potential to deny input and participation from the young people most relevant to study aims (i.e., the individuals with the suicide‐related risks that the interventions aim to support) [7]. Therefore, in addition to integrating community partner input into approaches for determining co‐designer readiness, guidelines that can be employed flexibly and account for collective decisions about readiness to participate that integrate teen, parent and therapist input are needed.

Finally, similar to other youth co‐designer concerns about researchers ‘ticking the box’ in participatory co‐design [33], co‐designers in the present case study identified the need to ensure that procedures are implemented in a way that is meaningful to them. Moreover, co‐designers recommended that researchers find ways to connect by utilising their preferred communication methods (e.g., texting as opposed to phone calls). Planning for maintaining secure communications that align with teen norms will be important for maintaining communication.

### 4.2 Co‐Design Engagement

As Michail et al. (2023) described, ‘safety within the context of suicide prevention research should also be taken to mean how comfortable and able a young person is to fully draw on their experience to inform the research they are involved in’ [6, p. 4]. Co‐designers identified the need for improvements that mirror those described by other youth‐involved researchers: the balance between prioritising social experiences, addressing emotional experiences, and considering cognitive barriers [33]. For example, the social components of each meeting (i.e., ice breakers and other rapport‐building activities) were viewed differently by participants according to their individual goals, pointing to the benefit of splitting partners into smaller groups not only based on age/cognitive abilities, but also based on personal goals. Some co‐designers were eager to immediately contribute to content development, while others appeared more interested in socialising.

Although our facilitation approach aimed to share power and ownership of topics to address with adolescent partners, the structured format helped set an agenda for adolescents to be able to just ‘show up’ and engage at a level appropriate to them. Striking this balance, allowing for flexibility in activities and discussion points without burdening participants to lead themselves, was difficult to achieve. While the added safety concerns related to monitoring distress and considering suicide‐related risk added to the complexity of this facilitation approach, key difficulties related more generally to holding adolescents' attention and encouraging balanced discussion across participants, mirroring the difficulties of group facilitation with youth, irrespective of suicide‐related histories, more generally. Given that researchers and funders are increasingly calling for Patient and Public Involvement and Engagement in the development and evaluation of mental health interventions [6, 7, 8, 34], it is especially critical that researchers sensitively and thoughtfully prepare for such workshops, given the difficult nature of facilitating active involvement in youth participants [7].

### 4.3 Implications

Co‐designers described the importance of integrating teen perspectives into intervention research and also the need for researchers and teens to prepare for the experience. The benefits of participatory co‐design for people with lived experience and researchers, and also the need to acknowledge that the experience can involve risk [6] and take a substantial amount of time, resources and expertise [35], are well described. As researchers continue to prioritise partnerships with individuals with suicide‐related risk, they must attend to the development and refinement of safety protocols appropriate for their partners. Researchers can leverage existing guidelines, especially those developed based on the insight of researchers and young people with lived experience [22, 23], and then iteratively refine them based on community partner perspectives and the individual strengths and needs of young people with lived experience.

Building on existing guidance regarding safety when engaging in co‐design with young people with lived experience [22, 23, 36], our findings contribute additional considerations for safely partnering with teens with previous suicide‐related crises. First, instead of limiting who can and cannot participate based on the timing of crises, it may be important to consider readiness for different aspects of the design. As we learned with our co‐designers, readiness may not be easily categorised based on the timing of a crisis, and it may fluctuate over time. Additionally, some of the safeguards themselves can prevent access to participation for marginalised youth. Our community advisors differed in their recommendations for requiring participants be in therapy for this reason—as specific racial and ethnic groups may be less likely to engage in therapy due to stigma and/or obstructions to treatment [37, 38]. Reviewing the different aspects of participation with each teen and a family member or supporting clinician allows for an inclusive approach that can also maintain teen safety.

Second, researchers should provide explicit examples of the type of content that is and is not appropriate to discuss. While the range of this content depends on one‐on‐one check‐ins with each co‐designer in advance of group sessions, researchers should be careful to evaluate their own assumptions about the appropriateness of certain topic areas. Youth with recent suicide‐related crises may benefit from discussions of shared experiences related to recovery, as long as discussions maintain boundaries regarding explicit content that could provoke distress (e.g., means for suicide and violence). Such conversations may be especially important for ethnic‐racial minoritised and LGBTQ+ youth, given the discrimination, homonegativity and anti‐queerness they may face in their daily home and school environments [39] and the protective role of connectedness against STB.

Third, we recommend that researchers not only evaluate involvement (e.g., soliciting feedback from young people about workshops and assessing the impact of participation on youth well‐being [23]) but also evaluate safety protocols. Similar to the present case study, researchers may consider reviewing safety protocols with co‐designers and integrating co‐designer feedback (when appropriate) into their approach.

## 5 Conclusion

Despite increased interest in participatory co‐design, few studies have described safety protocols used with adolescents with suicide‐related experiences under the age of 18 or focused specifically on ethnic‐racial minoritised or LGBTQ+ individuals. This case study provides an overview of the development and use of safety protocols for participatory co‐design with adolescents identifying as LGBTQ+ and/or ethnic‐racial minoritised with histories of suicide‐related crises. No safety issues emerged during co‐design sessions, and adolescent co‐designers endorsed many components of the safety protocol, with recommendations for improvements mainly addressing the need to adapt the protocol based on the unique needs of each teen. A key next step in this type of work is to move beyond exclusive foci on risk reduction towards a balanced consideration of risk reduction and a strengths‐based approach, acknowledging and leveraging the strengths and resilience of young people with STB. As described by our participants, youth with lived suicide‐related experience are often resilient and strong, with a ‘tolerance’ for handling complex challenges.

## Author Contributions

**Marisa E. Marraccini:** study conception and design, funding acquisition, methodology, data coordination, project administration, supervision, formal analysis, writing – original draft, writing – reviewing and editing. **Telieha J. Middletona:** data curation, formal analysis, writing – review and editing. **Lauren E. Delgatya:** data curation, formal analysis, writing – review and editing. **Ceren E. Iz:** data curation, software, writing – review and editing.

## Disclosure

The content is solely the responsibility of the authors and does not necessarily represent the official views of NIH, MQ or AFSP.

## Ethics Statement

Informed consent/assent, as well as parental consent, has been appropriately obtained. Study procedures were reviewed and approved by the University of North Carolina at Chapel Hill Institutional Review Board (#23‐0388). Initial approval was obtained on 06/19/2023 and was most recently renewed on 04/04/2025.

## Consent

Informed consent and/or assent were obtained from all individual participants included in this study.

## Conflicts of Interest

The authors declare no conflicts of interest.

## Supporting information


**Supplementary Table 1:** Final Safety Protocol for Participatory Co‐Design with Youth with Suicide‐Related Crises. **Supplementary Table 2:** Demographic Measures.

## Data Availability

As per ethical requirements, participant data will remain confidential. Anonymised data can be shared upon reasonable request to the primary author.

## Endnotes

1 Although we first engaged in co‐design sessions with adolescents while they were receiving treatment for a suicide‐related crises in an inpatient facility and subsequently completed individual meetings with adolescents who sought emergency services for a suicide‐related crisis to sense check co‐designer ideas, safety protocols and procedures for co‐design activities with these groups of participants were comparable to traditional safety protocols and thus are not the focus of this case study.
