# Supplementary material for: Partnering With Teens With Past Suicide‐Related Crises: Development, Application and Refinement of Safety Procedures for Co‐Design
Source: Health Expect. 2026 Feb 9;29(1):e70520. doi: 10.1111/hex.70520 (PMC12885166; doi:10.1111/hex.70520)
Supplement: Supplementary file 1 — Supplementary Table 1: Final Safety Protocol for Participatory Co‐Design with Youth with Suicide‐Related Crises. Supplementary Table 2: Demographic Measures. [file HEX-29-e70520-s001.docx]

**Partnering with Teens with Past Suicide-Related Crises: Development, Application, and Refinement of Safety Procedures for Co-Design**

Supplementary Table 1

Final Safety Protocol for Participatory Co-Design with Youth with Suicide-Related Crises

| Stage: Consideration | Description |
| --- | --- |
| **Preparation** |  |
| Community Partner Feedback | Our initial draft of the protocol (including participant eligibility criteria) was reviewed by members from relevant, local organizations to ensure we were prioritizing and considering the cultural context of co-designers in the co-design process. Because this work focuses on partnerships with racial or ethnic minoritized youth and youth identifying as LGBTQIA+ with a history of hospitalization for suicide-related crises, we sought feedback from local therapists and other providers (*n*=6) who have worked with adolescents with suicide-related thoughts and behaviors and specifically ethnic and racial minoritized adolescents and adolescents identifying as LGBTQ+. |
| Eligibility Criteria to Enhance Safety | Adolescent eligibility criteria for Aim 1 (co-design phase) of the study:   1. emergency department encounter for suicidal thoughts or behaviors in the past 2 years, with most recent hospitalization at least 6 months ago; 2. ages 13-19; 3. ability to speak, read, and understand English sufficiently to complete study procedures; 4. consent of a parent/legal guardian (provided in English or Spanish); 5. adolescent assent; 6. self-identifies as (a) minoritized race or ethnicity (e.g., Black or African American, Hispanic or Latino/a/e) and/or (b) minoritized sexual or gender identity (e.g., LGBTQ+); 7. Engagement in therapy or counseling and authorization to share information with the therapist/counselor. Consider getting clearance from the therapist regarding participation as appropriate. If at least a year has passed since most recent crisis, adolescent may participate without current therapist if adolescent and adult agree they are in a good place to participate.   Exclusion Criteria includes current psychotic symptoms and intellectual disability (mild to .profound). Risk for developing cybersickness would exclude VR participation, but not participation in co-design procedures. |
| Recruitment Process | - A minimum of two contacts with adolescents will occur prior to group interactions: a recruitment overview and an individual meeting. - We will clearly specify the lived experiences required to participate: emergency encounter for suicide-related thoughts and behaviors between 6 months and 2 years ago. - During recruitment, we will discuss readiness for research, including the question: Are you in a place in your recovery where this would be healthy? Per above, if at least a year has passed since most recent crisis, adolescent may participate without current therapist if adolescent and adult agree they are in a good place to participate. - Remind families that research is not a replacement for professional support. Reinforce importance of ongoing treatment and psychological care. |
| **Meeting Format** | - All participants will complete a one-on-one meeting with the researcher(s) prior to attending any group meetings to build rapport, address stressors, and collaborate on a safety plan. - Group sessions will include at least two trained researchers/clinicians. - As possible, separate groups based on age and identity (e.g., children ages 13-15 could attend a different group than 16-19). |
| Introductory Meeting | An individual with clinically trained staff member is required prior to group participation. The meeting will involve:   - Review of procedures (sensitive topics, potential stressors); - Discussion of any stressors to be aware of; - Discussion of why researchers are interested in working with minoritized groups to increase trust and understanding - Collaboration on safety plan to use as needed during sessions (both teen and researcher maintain copies). If a safety plan is already in use, adapt as needed for participation. If no safety plan has been developed, build one together.   - Review and adapt the safety plan any time there is a break between participation or as needed   - Safety plan will be adapted from Stanely & Brown to include:     - Details of anything person might find difficult or distressing     - Preferred contact method (adhering to UNC constraints around not texting minors)     - Updates after each use based on what was helpful     - Overview of steps taken if person appears at risk to harm of self or others     - Name and number of contact person on research team     - Motivations to live, future goals, protective factors - Agreement on Subjective Use of Distress Scale (SUDS) that identifies where on scale participants will employ calming strategy (template to be shared should be colorful and visually appealing); - Gather information on calming strategies they use that can be offered during group - Review confidentiality, including examples of when confidentiality would be broken and the types of information to be shared to therapists. - Review aim to have a private space for group sessions; if private space unavailable, brainstorm ways to increase privacy (e.g., headphones). Also remind adolescent (if 17 or under) that they need to have an adult home with them during sessions in another room). - Explain use of breakout rooms and anonymous feedback forms during sessions so teens know how they will be used. Discuss reliable self-generated ID code (use favorite color and favorite fictional character – e.g., blue mickey). Then create and record it together. - Identify how teen wants to be introduced (discuss preferred pronouns and safety of using them in virtual meeting if others are at home). - Plan for access to virtual meetings (provide Chromebook as needed) and plan for connectivity disruptions (collect best number to reach out to should someone be dropped) - Gather multiple forms of contact in case of emergency; - Share resources as needed - Share “Review Tips” from *Guidelines for Involving Young People with Lived and Living Experiences of Suicide in Suicide Research* (pp18-19). |
| Group Meetings | The format of the group meetings will include the following:   - Prior to the start of the meeting, display a warm-up activity that will not be shared with the group to get their creativity flowing and allow for space to feel comfortable when entering group - Check-in/check-out: at the start and conclusion of each session, participants rate SUDS   - If SUDS is at or above individual’s threshold for concern, have second person available for immediate check in; - Conducted in safe, private space; if online, be sure to encourage young people to find a safe, private space at their home/environment; review considerations as needed in cases when privacy is not possible. Ensure that there is an adult home with all adolescents (17 and under). - Allow access to breakout rooms anytime someone wants a break; prepare for need by ensuring a clinician will head to a breakout room should a participant go there. - Each meeting will include a content warning ahead of time, with explicit overview of structure of involvement and any preparation needed, and any potential stressors involved; - Always remind people they can choose not to share; they can step away as they wish and need and to let a researcher know; they can change their level of involvement; it can be normal to feel uncomfortable; - Always review confidentiality, and limits of confidentiality (individual – risk of harm to self or others; and group – cannot ensure others won’t share discussions); provide concrete examples - Researchers will not share their own lived experiences in these meetings; - Recordings are paused during introductions, safety planning, and other confidential discussions; during introductions, ask all participants to state preferred name and pronouns for others to use - Provide calming activities and sensory activities; - Group cool downs at end of session: reflect on learning and challenges, allow to choose one-on-one and group reflection and group activity, encourage self-care after experience. Remind participants that self-care means different things to different people and it can be difficult depending on individual circumstances. Normalize and validate. - In case of connectivity issues/internet access issues: a researcher will make two attempts to reach out to participant via another contact method (e.g., phone) if connection is not re-established during meeting to ensure safety and check-in. Spanish speaking research assistants will be on call if they are not attending the meeting in the event that a Spanish speaking family needs to be contacted. Researchers will problem solve with families around providing access to borrowed Chromebooks or hotspots if access to devices/internet service is a barrier to participation. |
| Initial Group Meeting | - First meeting come to consensus on group rules, which include a minimum of:   - Keeping everything confidential, respect others privacy and do not repeat discussions;   - No sharing of contact information;   - No sharing particularly graphic or stress-inducing anecdotes/stories/discussions/images, including (note that we will encourage students to reach out to us individually if they have specific concerns about off limit topics):     - Sexual interactions, nudity, or sexual abuse     - Details of self harm or suicide thoughts and behaviors     - Drug or alcohol use     - Abuse or neglect     - Dating violence     - Specific details of trauma experiences   - Identify what a safe environment looks like;   - Clarify that the goal of this is not to share lived experiences of suicide, invite them to discuss what they do and do not want to share;   - Remind everyone to respect one another’s boundaries, and to be respectful of shared lived experiences;   - Encouragement to monitor mood and take breaks as needed;   - Remind members about taking turns talking, no interrupting - Clearly communicate clinician’s role – support and scaffolding for researchers and participants - Provide training around:   - Diversity of lived experiences with suicide-related thoughts and behaviors   - How to safely communicate about suicide   - Processes for conducting ethical suicide research   - Taking care of yourself |
| Check-Ins Between Meetings | - Reach out to youth to check-in about safety and wellbeing within a week of first, middle, and last meeting using preferred method:   - Ask for their experiences, questions, and feedback;   - Inquire about well-being overall. - If concerned, follow risk protocols and procedures and consider sharing information with family/clinician - If non-responsive, try other methods of contact (ensure we have more than one ahead of time). |
| **Evaluations** | Researchers will provide co-designers an opportunity to provide formal feedback and also evaluate efforts through observations and processes throughout:   - Feedback provided throughout processes; - Observations conducted during processes; - Distress levels provided before and after sessions; - Debriefs at end of co-design with researchers and partners to evaluate:   - Level of distress in involvement;   - Any positive outcomes;   - If they need to speak further with researcher or clinician;   - Ways to improve involvement;   - Comfort in discussing experiences. |
| **Safety & Wellbeing Protocols** | - **How to respond to distress:** Refer to participant’s safety plan and the calming strategies provided at the end of this manual and engage in one-on-one session. Connect with family member and/or clinician as needed. - **How to react if youth share suicidal thoughts and behaviors:** Provide safe, one-on-one setting to conduct risk assessment procedures as needed, consult with lead clinician, connect with family member and/or clinician. - **How to respond to an attempt:** Conduct risk assessment, connect with family member and/or clinician, consult with lead clinician, and refer for services as needed. - **Minimization of risks:** Combination of one-on-one virtual and in-person meetings, and structured group Zoom meetings - **Roles:**   - **Lead PI/Clinician:** [NAME]   - **Clinical Extern:** [NAME]   - **Research Staff:** [NAME] - **Confidentiality Exceptions:**   - If we are concerned that you or someone else may be in danger, we will need to share this information with a family member and/or your clinician. Spanish speaking research team member will proactively practice how to speak to parents about safety concerns or dropped connections before meetings start so they feel comfortable. - **Safety Signals:** Allow for a range of communication methods to indicate pausing, stepping away, opting out: emojis, safety words, hand signals. - **Anonymous Reporting Form:** For 30 minutes before and after meetings as well as throughout the meeting time, an anonymous reporting form will be available for participants to note concerns regarding safety or other issues that they’d like to discuss. Forms will not be anonymous for clinicians but will be anonymous for other group members. - **Calming Strategies:** The research team will have calming strategies (e.g., sensory awareness, muscle relaxation) available to support co-designers as needed. |

Supplementary Table 2

Demographic Measures

| Demographic Variable | Measure Description |
| --- | --- |
| Race | Race was first measured with the following question: “Which of the following describes you (Check all that apply)?” Response options were: American Indian or Native Alaskan, Asian, Black or African American, Native Hawaiian or Other Pacific Islander, White, Other, and Prefer not to answer. Race was also measured with an additional question: “When thinking about physical attributes usually ascribed to race, which of the following general labels describe how you/others would describe yourself racially (mark ALL that apply)?” Response options were: Asian, Black, Aboriginal or First Nations, Latino or Hispanic, Middle Eastern, White, Other, and “I prefer not to answer” (Hughes et al., 2022). Ethnicity was measured with the following question: “Are you Hispanic or LatinX?” Response options were: Yes, No, and Prefer not to answer. |
| Gender Identity | Questions asking about gender and sex were informed by recommendations from the GenIUSS Group (Badgett et al., 2014). Physical sex was measured by asking “What physical sex were you assigned at birth (what the doctor put on your birth certificate)?” with responses including Male, Female, or Intersex. Gender was measured with the following question: ‘When a person’s sex and gender do not match, they might think of themselves as transgender. Sex is what a person is born with. Gender is how a person feels. What one response best describes you?’. Response options were: “I identify as a boy or man,” “I identify as a girl or woman,” “I identify in some other way,” “I do not know what this question is asking,” and “I do not know if I am transgender.” |
| Sexual Orientation | Sexual orientation was measured by asking: “What do you consider yourself to be?” Response options were: heterosexual or straight, gay or lesbian, bisexual, pansexual, and other. |

Supplementary References

Badgett, M. L., Baker, K., Conron, K., Gates, G., Gill, A., Greytak, E., & Herman, J. (2014). Best practices for asking questions to identify transgender and other gender minority respondents on population-based surveys (GenIUSS). *The GenIUSS Group, UCLA Williams Institute*.

Hughes, J. L., Camden, A. A., Yangchen, T., Smith, G. P., Domenech Rodríguez, M. M., Rouse, S. V., McDonald, C. P., & Lopez, S. (2022). Guidance for researchers when using inclusive demographic questions for surveys: Improved and updated questions. *Psi Chi Journal of Psychological Research*, *27*(4), 232–255.
